# Serum glial fibrillary acidic protein in acute stroke: feasibility to determine stroke-type, timeline and tissue-impact

**DOI:** 10.3389/fneur.2024.1470718

**Published:** 2024-12-06

**Authors:** Julien F. Paul, Célina Ducroux, Pamela Correia, Audrey Daigneault, Catherine Larochelle, Christian Stapf, Laura C. Gioia

**Affiliations:** ^1^Department of Neurosciences, University of Montreal, Montréal, QC, Canada; ^2^Division of Neurology, Department of Medicine, Centre Hospitalier de l’Université de Montréal (CHUM), Montréal, QC, Canada; ^3^Neurosciences Axis, Centre de Recherche du CHUM (CRCHUM), Montréal, QC, Canada; ^4^Division of Neurology, The Ottawa Hospital, Ottawa, ON, Canada

**Keywords:** ischemic stroke, intracerebral hemorrhage, biomarkers, GFAP, large-vessel occlusion

## Abstract

**Background:**

Interest is emerging regarding the role of blood biomarkers in acute stroke. The aim of this pilot study was to determine the feasibility of biomarker acquisition in suspected acute stroke, using modern ultrasensitive immunoassay techniques, and explore their potential usefulness for stroke diagnosis and management.

**Methods:**

In 62 patients with suspected acute stroke, blood samples were prospectively obtained upon arrival and prior to neuroimaging. Serum levels of glial fibrillary acidic protein (sGFAP) and neurofilament light chain (sNfL) were analyzed using a single molecule array (SIMOA^®^) method, according to time of symptom onset, neuroimaging, and final diagnosis.

**Results:**

Acute ischemic stroke (AIS) was diagnosed in 35 patients, 10 with large-vessel occlusion (LVO). The remaining were diagnosed with intracerebral hemorrhage (ICH) (*n* = 12), transient ischemic attack (*n* = 4), and stroke mimics (*n* = 11). Median (IQR) sGFAP levels were significantly higher in ICH (2,877.8 [1,002.1–10,402.5] pg./mL) compared to others diagnoses. In AIS, GFAP levels appear to increase with longer delays since symptom onset and were higher in patients with more extensive ischemic changes on baseline CT (ASPECTS ≤7) than those without, particularly in LVO stroke. NfL values were similar across groups.

**Conclusion:**

In acute stroke, serum GFAP levels show potential as an adjunct tool for the distinction between ICH and AIS. Specific to AIS, GFAP may also offer insight into time from onset, and extent of ischemic tissue injury on neuroimaging, particularly in LVO stroke. These preliminary findings merit further study.

## Introduction

Current management of acute ischemic stroke (AIS) and intracerebral hemorrhage (ICH), requires (1) neuroimaging to provide a diagnosis and (2) a time of symptom onset to determine eligibility for treatment. In up to 30% of patients, however, time of symptom onset is either unknown or exceeds recommended time windows for treatment (1). Advanced neuroimaging biomarkers are now widely recognized key criteria in acute stroke management, independent of time, to identity eligible patients (2). Accordingly, a paradigm shift is evolving away from time-based decision algorithms and toward physiology-based acute stroke management strategies. By extension, brain-specific blood biomarkers may offer an innovative opportunity to provide physiology-based information and potentially improve accessibility to acute stroke treatments.

Glial fibrillary acidic protein (GFAP), an astrocytic protein found almost exclusively in the brain, is a promising biomarker of brain tissue damage in neurological conditions, including stroke (3). In the first 6 h, GFAP can discriminate between AIS and ICH (4), presumably due to acute brain damage incurred following hematoma formation and the immediate disturbance of the brain blood barrier in ICH compared to AIS where there is a slower transition between penumbral tissue into core over time in the absence of reperfusion (5). Neurofilament light chain (NfL), a novel biomarker for axonal injury, shows promise as a prognostic tool following AIS (6). However, data regarding its use in acute stroke is limited. In most studies, GFAP was measured using ELISA techniques (expressed in ng/mL) (7) or did not include stroke patients beyond the hyperacute phase (8), thus limiting information in acute stroke of unknown or delayed onset. The aim of this pilot study was to evaluate the feasibility of blood biomarker acquisition upon Emergency Department (ED) arrival in suspected acute stroke using Single Molecule Array (SIMOA), a novel ultrasensitive immunoassay. In addition, we conducted exploratory analyses to compare biomarker levels according to time of symptom onset, acute neuroimaging, and final diagnosis.

## Methods

### Study design

In this single-center prospective observational, descriptive pilot study, patients evaluated for a suspected acute stroke <24 h from known symptom onset or last seen well were recruited over 6 months at our comprehensive stroke center.

### Sample collection and storage

Blood samples were obtained during routine blood draw upon ED arrival. After 30 min to allow for coagulation, samples were centrifuged at 1800 g for 10 min at 15–24°C to separate cells and serum. Serum was then frozen at −80°C in aliquoted cryotubes until analysis.

### SIMOA analysis

Serum GFAP (sGFAP) and serum NfL (sNfL) levels were measured in duplicates with the SR-X detection system using the SIMOA Neurology 2-Plex B Kit (Quanterix, Billerica, MA, USA). Analysis was carried out according to the manufacturer’s instruction. Briefly, SIMOA is a highly-sensitive multiplex technique using paramagnetic antibody-coated microbeads specific to GFAP and NfL, which emit a signal if detected in patient serum by immunofluorescence which is then converted digitally for bead (and biomarker) quantification (9).

### Data collection

Clinical data was collected from a data repository of all acute stroke patients evaluated at our center, where we extracted the following variables: age, sex, relevant past medical history (previous stroke, epilepsy, or cognitive disorders), clinical stroke scales [National Institutes of Health Stroke Scale (NIHSS), modified Rankin Score (mRS)] and variables of interest including time of stroke onset, time of ED arrival, neuroimaging data presence of ICH or early ischemic changes as per the Alberta Stroke Program Early CT Score (ASPECTS), and presence of large-vessel occlusion (LVO) on CT angiography, final diagnosis. Time to blood acquisition and centrifugation were also recorded as part of study procedures.

### Statistical analyses

Variables were reported as mean ± standard deviation (SD), median and interquartile range (IQR) or proportions (%) as appropriate. Due to skewed data distribution, sGFAP and sNfL values were compared across final diagnosis group using the Kruskall-Wallis test, followed by Dunn’s post-hoc test for pairwise comparisons. Correlation between time of stroke onset and sGFAP values was assessed using the Spearman’s rank correlation coefficient (*ρ*), stratified by LVO presence. Comparisons between unfavorable (≤7) and favorable (8–10) ASPECTS were performed using the Wilcoxon rank-sum test. To examine potential interactions between time, ASPECTS, cognitive disorder or previous stroke and sGFAP levels, linear regression analyses were conducted, with sGFAP values log-transformed to address skewness in the data distribution.

## Results

Blood samples at ED arrival were collected in 68 patients with suspected acute stroke. Of these, 6 were excluded due to delays in sample processing. Among the remaining 62 patients, median (IQR) time from ED arrival to sample acquisition was 11 (9–24) minutes and 48 (37–70) minutes from sample acquisition to centrifugation. Regarding biomarker analyses, intra-assay variabilities (coefficient of variation, CV, in %) for duplicate measures was 6.2% (sGFAP) and 6.9% (sNfL); 88% of samples had intra-assay CV <20%.

Baseline characteristics are summarized in Table 1. AIS was diagnosed in 35 patients, of which 10 (29%) had a large-vessel occlusion (LVO). ICH was diagnosed in 12, transient ischemic attack (TIA) in 4, and stroke mimics in 11, where stroke mimics included seizures, functional neurological disorder and brain tumor (1). Median time from symptom onset to sample acquisition was 101 (73–149) minutes in the whole study population. Among the 12 AIS patients with known time of stroke onset, median time from stroke onset to sample acquisition was 91 (55–118) minutes. Median time from last seen well to sample acquisition was 738 (297–917) minutes in the 23 patients with stroke of unknown onset.

**Table 1:** Characteristics of study population.

Study population	62
Age, years (mean ± SD)	68.5 ± 16.5
Female sex, *n* (%)	27 (44)
Past Medical History *n* (%)	
Previous Stroke	14 (23)
Intracerebral Hemorrhage	2 (3)
Epilepsy	3 (5)
Neurocognitive Disorder	9 (14.5)
Modified Rankin Score, median(IQR)	1 (0–1)
Initial NIHSS, median(IQR)	9 (3–17)
Sample processing delays, minutes (median(IQR))	
ED arrival to sample acquisition	11 (9–24)
Symptom onset to sample acquisition	101 (73–149)
Last seen well to sample acquisition	683 (287–917)
Sample acquisition to centrifugation	48 (37–70)
By final diagnosis	
(A) Acute Ischemic Stroke	35
LVO Stroke	10
ASPECTS, median(IQR)	10 (9–10)
ASPECTS ≤7	5 (14)
Known stroke onset, n (%)	12 (34)
Time, stroke onset to ED arrival	63.5 (46–90)
Time, last seen well to ED arrival	698 (282–861)
(B) Intracerebral Hemorrhage	12
Known onset	7 (58)
Time, onset to ED arrival	90 (66–99)
Time, last seen well to ED arrival	329 (185–350)
(C) Transient Ischemic Attack	4
(D) Stroke Mimics	11

ASPECTS, Alberta Stroke Program Early CT Score, ED, Emergency Department, NIHSS, National Institutes of Health Stroke Scale.

**Figure 1:**
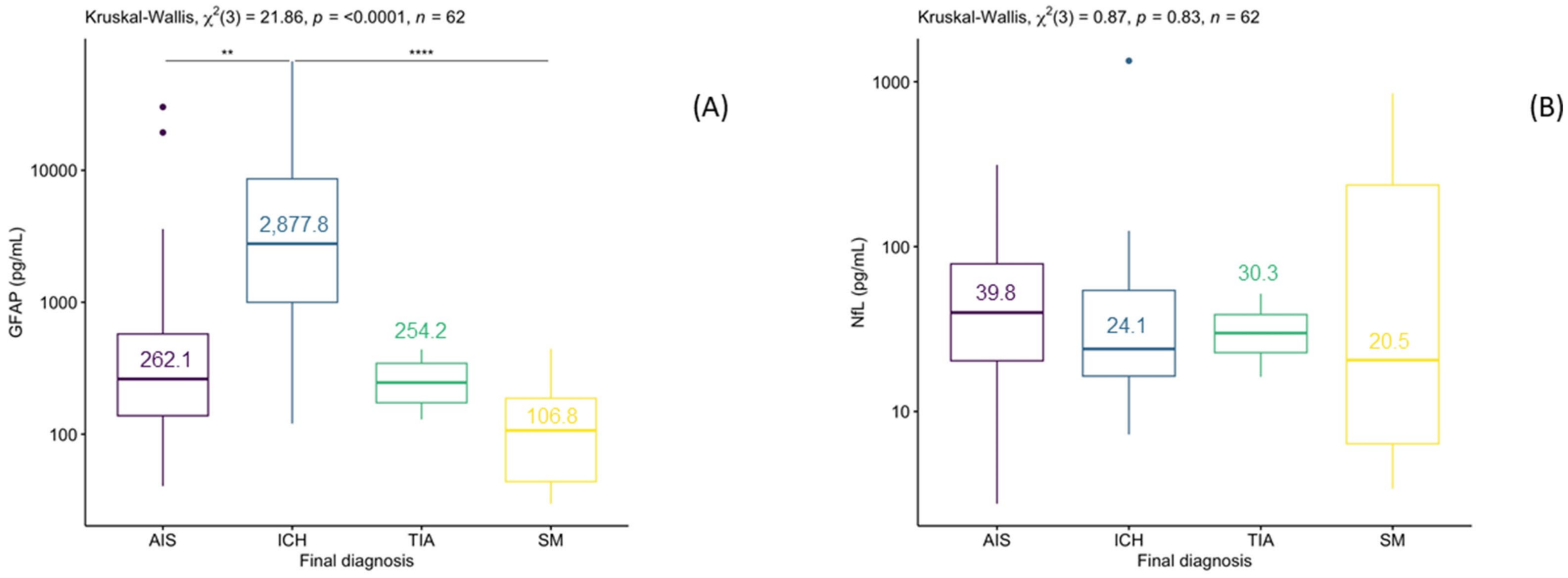
Box plot of median sGFAP values **(A)** and sNfL **(B)** values (pg/mL) in patients presenting with suspected stroke, classified according to final diagnosis.

Acute stroke patients (AIS or ICH) had higher median sGFAP concentrations (384.9 [164.3–1.576.6] pg./mL) compared to those with TIA (254.2(175.4–348.1 pg./mL) or stroke mimics 106.7 (44.9–207.5 pg./mL) *p* < 0.001), with ICH showing highest median sGFAP levels (2,877.8 [1,002.1–10,402.5] pg./mL) (Figure 1A). sNfL levels did not differ between groups (ICH [24.1(16.4–54.3) pg./mL]), AIS (39.8 [20.3–78.6] pg./mL), TIA (30.3(23.2–39.3) pg./mL), stroke mimics (20.5(6.4–379.8) pg./mL) (Figure 1B).

Five out of 35 AIS patients reported a history of cognitive disorders. Linear regression analysis revealed no significant interaction between cognitive decline or age and sGFAP levels. Additionally, 6 AIS patients had a history of previous stroke. Regression analyses revealed a positive interaction exists between prior stroke and sGFAP levels. (Supplementary Table S1) However, the presence of a prior stroke was associated with lower sGFAP levels. No AIS patients had a history of epilepsy.

**Figure 2:**
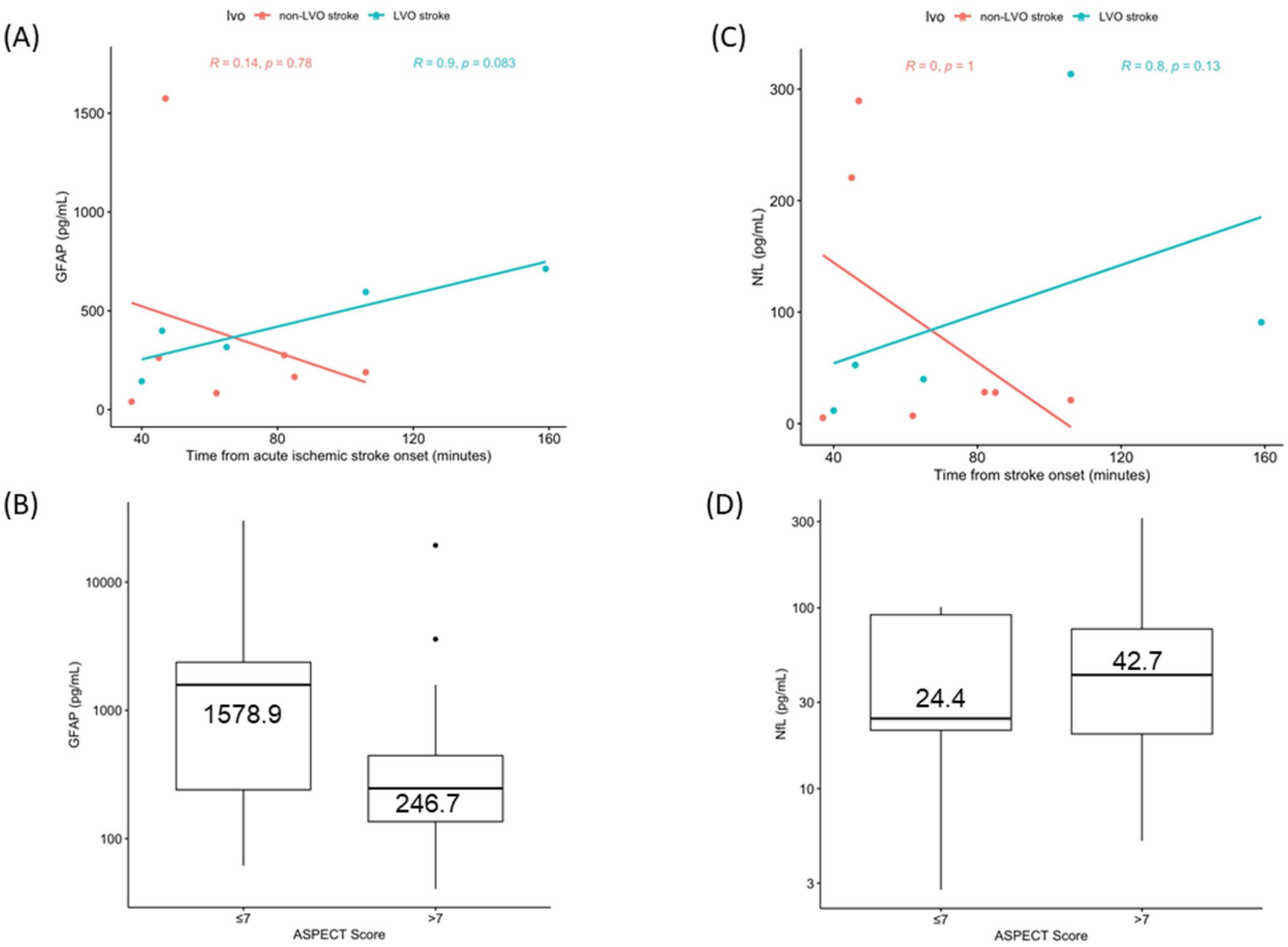
Scatterplots with regression lines of sGFAP **(A)** and sNfL **(C)** in Acute Ischemic Stroke with known onset, stratified for LVO presence. Boxplot of sGFAP **(B)** and sNfL **(D)** according to ASPECTS.

Among AIS patients with known time of stroke onset <4.5 h (*n* = 12), sGFAP levels appeared to increase with longer delays from symptom onset, particularly among the 5 patients with LVO stroke (*ρ* = 0.9, *p* = 0.08) (Figure 2A), but was not observed among those with stroke of unknown onset (*ρ* = 0.39, *p* = 0.07). No correlation was observed between sNfL levels with time from stroke onset, in both LVO (*R* = 0.8, *ρ* = 0.13) and non-LVO subgroups (*ρ* = 0, *p* = 1) (Figure 2C).

Regarding neuroimaging, all 12 patients with known stroke onset presented with favorable ASPECTS (8–10), and none with ASPECTS ≤7. Among the 23 patients with unknown time of stroke onset and > 4.5 h from last seen well, 5 had an ASPECTS score ≤ 7. Median sGFAP levels were higher in AIS with ASPECTS ≤7 (*n* = 5) (1,578.9(239.8–2,370.5) pg./mL) than those with ASPECTS 8–10 (*n* = 30) (246.7(135.5–444.5) pg./mL) (*p* = 0.2) (Figure 2B). Median sNfL levels were similar in ASPECTS ≤7 (24.4(21.0–91.6) pg./mL) and ASPECTS 8–10 (42.7(20.1–76.4) pg./mL) (*p* = 0.87) (Figure 2D). Regression analysis revealed no significant interaction between time from last seen well, ASPECTS, and GFAP levels in stroke of unknown onset. (Supplementary Table S1).

**Figure 3:**
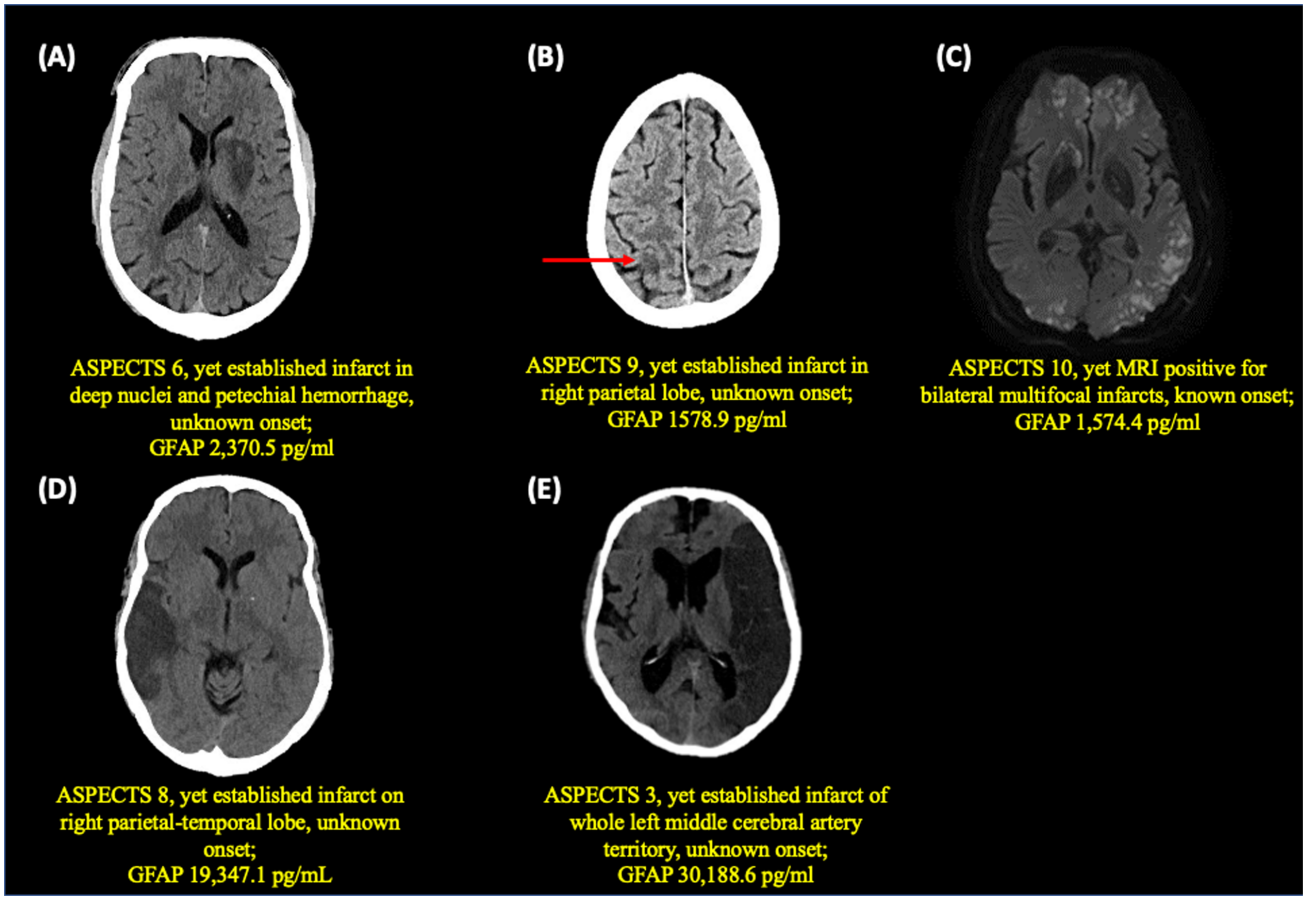
Baseline neuroimaging in 5 patients **(A–E)** with highest sGFAP values (>1,000 pg./mL).

Of note, 3 patients had higher sGFAP levels despite favorable ASPECTS. In detail, one of these patients presented with stroke of known onset and an ASPECTS 10 on baseline non-contrast CT had a markedly elevated sGFAP level (1574.4 pg./mL). However, MRI imaging demonstrated an embolic shower of innumerable millimetric DWI lesions. (Figure 3C). Among the other 2 patients, both presented with stroke of unknown onset. Despite being classified as ASPECTS scores of 8 and 9 respectively, both patients had consolidated hypodense infarcts (in contrast to more subtle early ischemic changes) (Figures 3B,D).

## Discussion

Brain-specific blood biomarker acquisition and analysis is feasible in acute stroke and shows promise as an adjunct tool in acute stroke management. In our study, sGFAP levels were markedly higher in acute ICH than AIS using a modern ultrasensitive immunoassay (SIMOA), consistent with previous published studies (7, 10). In exploratory analyses, we also found that among patients with AIS of unknown onset, median GFAP levels were higher in those with more extensive changes on baseline neuroimaging. Furthermore, among patients with known time of stroke onset, serum GFAP levels appear to increase with longer delays since symptom onset, particularly in LVO stroke.

To our knowledge, this is the first study assessing sGFAP levels in the emergency setting of acute stroke that included stroke of unknown onset beyond 4.5–6 h. In this subgroup, no clear trend was observed between time from last seen well and sGFAP levels, likely due to the heterogeneity of the population of stroke patients with unwitnessed stroke onset (including both those with prolonged symptoms and those with a more recent onset). Among patients with known time of stroke onset, we observed a linear relationship between sGFAP levels and time, particularly in LVO stroke. However, given the small sample size and the absence of serial measurements of GFAP over time in each patient, these findings should be interpreted with caution and are meant to be hypothesis-generating only. Nevertheless, these findings raise hypotheses regarding the potential role of sGFAP as a surrogate marker for ischemic injury over time in acute stroke, particularly in the context of evolving change of ischemic core into penumbra over time in LVO stroke. A recent study of GFAP acquisition in the early phase of stroke onset also observed that blood GFAP levels increases with time (11).

Secondly, we observed that higher median sGFAP levels were associated with unfavorable imaging (ASPECTS≤7). While GFAP release patterns in acute ischemic stroke are not yet fully understood, we and others hypothesize that more extensive parenchymal damage is associated with more astrocytic damage and higher disruption of the blood–brain barrier, resulting in higher GFAP release in the bloodstream (3). While these data are preliminary, they raise the question as to whether sGFAP levels could serve as a potential surrogate marker for ischemic brain injury in stroke of unknown onset. In our study, we did not observe a significant interaction between time, ASPECTS and GFAP levels. However, the results and their interpretation are limited given that time of stroke of onset is unknown in this subgroup, as well as a risk of model overfitting on account of the small number of subjects with low ASPECTS scores in the study. Nonetheless, this concept is of interest and warrants further investigation in larger studies to better understand the relationship between time, ASPECTS, and sGFAP levels.

Notably, we also observed elevated sGFAP levels in some patients despite a favorable ASPECTS scores. Further analyses revealed that despite “higher” ASPECTS scores (which generally indicate limited brain parenchymal damage), brain imaging revealed more consolidated or established infarcts in 2 patients and diffuse multifocal small infarcts on MRI not easily discernable on non-contrast CT in 1 patient. These cases underscore some of the limitations of ASPECTS in assessing the extent and evolution of brain infarcts on CT scan (12). At the same time, they also highlight the potential of brain-specific blood biomarkers, such as GFAP, to provide insight into the extent of brain tissue damage beyond standard neuroimaging, supporting their potential role as adjunct tools in acute stroke management.

This concept is of particular interest given emerging portable point-of-care technology, such as the i-STAT^®^ platform (13), able to measure plasma GFAP levels within minutes (14). Indeed, the need to develop portable tools to optimize acute stroke management was recently emphasized by the INTERACT-4 study assessing medical management in the ambulance in suspected yet undifferentiated stroke. In this study, intensive blood pressure reduction was associated with better outcomes in ICH yet showed harm in AIS, stressing the need to better differentiate stroke type in the ambulance (15). Current point-of care technology to assess GFAP levels requires centrifugation to obtain plasma, yet studies are ongoing to adapt this technology to the prehospital setting (16). Indeed, if rapid GFAP levels show promise as a surrogate marker for time and/or imaging in AIS of unknown onset, this could further optimize prehospital acute stroke management. Coupling GFAP levels with other inflammatory and/or cardiometabolic biomarkers could enhance detection of LVO stroke with favorable imaging, which in turn, could improve triage trajectories directly to comprehensive stroke centers with endovascular capabilities (17, 18), especially in resource-limited settings (19).

Given that elevated GFAP levels are observed across various neurological conditions, potential confounders may have influenced our results. To address this, we performed linear regression analyses examining the effects of cognitive disorder, age and prior stroke and sGFAP levels among AIS patients. Although a positive interaction was found between prior stroke and sGFAP levels, the presence of a prior stroke was associated with lower GFAP levels. This result is of uncertain significance given that the inverse association is counterintuitive and differs from previous studies (20), warranting further study in larger cohorts.

Contrary to sGFAP, sNfL has, to our knowledge, not been studied in the acute phase of undifferentiated stroke. However, in our study, we did not observe any significant associations between sNfL levels and diagnosis, time from stroke onset or neuroimaging.

## Limitations

Our study has several limitations. First, as a single-center observational pilot study with small sample size and no blinding, the findings are preliminary and should be considered hypothesis-generating only, requiring validation in larger cohorts. Additionally, the sample size may limit the power to detect meaningful associations, increasing the risk of type II error, and the possibility of model overfitting. Third, because the study was not designed as a diagnostic accuracy study, we were unable to calculate sensitivity, specificity of sGFAP to diagnose ICH.

SIMOA technology has increased sensitivity to measure blood biomarker levels (in pg./mL) when compared to conventional methods (21, 22). Its low detection threshold enables quantification of very low concentration of sGFAP and sNfL (16.6 pg./mL and 1.6 pg./mL, respectively) and has been increasingly applied in various neurological diseases (23). Nonetheless, SIMOA’s use in clinical practice is hindered by its high costs, complex procedures, and the need to process samples in batches, precluding its use to guide clinical management. Emerging developed point-of-care technology that can rapidly assess plasma GFAP levels could address some of the limitations of SIMOA going forward. Lastly, in our study, time from ED presentation to centrifugation was influenced by the 30-min wait required for blood coagulation. Newer assays that use plasma or ideally whole blood would eliminate this wait, and thus expediting GFAP analysis in the future.

## Conclusion

GFAP levels measured in blood is increasingly recognized as a promising biomarker in neurological diseases, including acute stroke. In our study, using SIMOA technology, we found that sGFAP levels are significantly elevated in ICH compared to AIS, consistent with previous research. Additionally, we observed that sGFAP levels are higher in patients with extensive ischemic injury, particular among those with stroke of unknown onset. With developing novel technology able to measure blood GFAP levels in minutes, the potential for GFAP to serve as an adjunct tool in acute stroke management, particularly in the prehospital setting, is increasing apparent and warrants further study.

## Data Availability

Aggregated data supporting the conclusions of this article can by made available by the authors upon reasonable request and according to institutional regulations.
