# Supplementary material for: Serum glial fibrillary acidic protein in acute stroke: feasibility to determine stroke-type, timeline and tissue-impact
Source: Front Neurol. 2024 Dec 6;15:1470718. doi: 10.3389/fneur.2024.1470718 (PMC11704488; doi:10.3389/fneur.2024.1470718)
Supplement: Supplementary file 1 [file Table_1.DOCX]

**Supplementary Table 1.** Exploring Serum GFAP levels in relation to a) cognitive decline, b) previous stroke, c) time and neuroimaging**.**

| **A. *s*GFAP levels and Cognitive Decline** | | | | | | | |
| --- | --- | --- | --- | --- | --- | --- | --- |
| **Predictor** | **B** | **Standard Error** | **ß** | **t** | **p-value** | **95% CI (lower)** | **95% CI (upper)** |
| (Constant) | 1.822 | 0.623 | - | 2.924 | 0.006 | 0.551 | 3.094 |
| Interaction cognitive decline & log GFAP | 0.843 | 0.803 | 1.138 | 1.049 | 0.302 | -0.795 | 2.481 |
| Age | 0.011 | 0.009 | 0.225 | 1.208 | 0.236 | -0.007 | 0.029 |
| Cognitive disorders | -2.333 | 1.941 | -1.292 | -1.202 | 0.238 | -6.292 | 1.625 |
| **B. sGFAP levels and Previous Stroke** | | | | | | | |
| (constant) | 2.529 | 0.104 | - | 24.208 | <0.001 | 2.316 | 2.742 |
| Interaction Previous_stroke & logGFAP | 1.000 | 0.288 | 1.632 | 3.475 | 0.001 | 0.414 | 1.586 |
| Previous stroke | -2.529 | 0.787 | -1.509 | -3.213 | 0.003 | -4.132 | -0.926 |
| **C. sGFAP levels and time and ASPECTS in stroke of unknown onset** | | | | | | | |
| (constant) | 3.630 | 1.403 | - | 2.588 | 0.19 | 0.67 | 6.589 |
| Time_LSW and ASPECTS | -4.045E-5 | 0.000 | -0.381 | -0.383 | 0.707 | 000 | 000 |
| Time_LSW | 0.000 | 0.001 | 0.476 | 0.458 | 0.653 | -0.001 | 0.002 |
| ASPECTS | -0.132 | 0.153 | -0.290 | -0.864 | 0.400 | -0.454 | 0.19 |

*sGFAP= serum glial fibrillary acidic protein, ASPECTS = Alberta Stroke Program Early CT Score, LSW= Last seen well
